# Bioabsorbable polymers in cancer therapy: latest developments

**DOI:** 10.1186/s13167-015-0045-z

**Published:** 2015-11-19

**Authors:** Ana C. Fonseca, Arménio C. Serra, Jorge F. J. Coelho

**Affiliations:** CEMUC, Department of Chemical Engineering, University of Coimbra, Rua Sílvio Lima-Pólo II, 3030-790 Coimbra, Portugal

**Keywords:** Cancer, Bioabsorbable polymers, Nanoparticles, Micelles, Implants

## Abstract

Cancer is a devastating disease, being responsible for 13 % of all deaths worldwide. One of the main challenges in treating cancer concerns the fact that anti-cancer drugs are not highly specific for the cancer cells and the “death” of healthy cells in the course of chemotherapy treatment is inevitable. In this sense, the use of drug delivery systems (DDS) can be seen as a powerful tool to minimize or overcome this very important issue. DDS can be designed to target specific tissues in order to mitigate side effects. Bioabsorbable polymers, due to their inherent characteristics, and because they can be synthesized in a variety of forms, are materials whose importance in the DDS for cancer therapy has risen significantly in the last years. This review intends to give an overview about the latest developments in the use of bioabsorbable polymers as DDS in cancer therapy, with special focus on nanoparticles, micelles, and implants.

## Review

### Introduction

Nowadays, cancer is the first cause of death in industrialized countries and the second cause of death in developing countries [1]. The disease was the cause of 7.6 million deaths worldwide, and 12.7 million new cancer cases are reported per year [2]. The most common types of cancer include breast, colorectal, prostate, and lung cancers being the last one, responsible for 1.6 million of the deaths [3]. Radiotherapy and surgery are the most used methods for the treatment of local and non-metastatic cancers, whereas chemotherapy is the main treatment for metastatic cancers. Often, the three treatments are combined together. Chemotherapy is based on the use of anti-cancer drugs that are intended to inhibit the fast proliferation of cancer cells, but inevitably, the lack of selectivity will damage healthy tissues causing adverse side effects. Additionally, due to their chemical structure, drugs also show low half-life times in the blood stream and a low bioavailability. These two facts often originate the need of higher drug dose administration, with concomitant problems related with undesired side effects [4–6]. In this sense, the use of drug delivery systems (DDS) can offer important “breakthroughs” in the field of chemotherapy. In general, a DDS allows the delivery of an active compound in a controlled way (time and release rate) and allows the maintenance of the drug concentration in the body within a more acceptable therapeutic window [7]. DDS can be formulated in the form of particles (microparticles, nanoparticles, micelles, liposomes) to be administered through the common routes (e.g., oral, pulmonary) or can in be used in the form of implants, both injectable (e.g., gels, microparticles) and surgical (e.g., sheets/films, foams, scaffolds) [8].

Polymers, both natural and synthetic, due to their versatility and characteristics have been widely used in the development of DDS. Natural polymers are highly biocompatible and biodegradable and present functional groups (e.g., -OH, -NH_2_) that can be easily modified. Synthetic polymers, in turn, have the possibility of being prepared with tailored compositions, and their properties can be easily adjusted, to match a specific application [9–11]. In particular, bioabsorbable synthetic polymers have particular relevance in the context of DDS since they (or their degradation products) can be metabolized in the biological environment [12].

This review intends to give an overview about the different DDS prepared using bioabsorbable polymers in the field of cancer therapy.

### Bioabsorbable polymers in DDS

Bioabsorbable polymers can be defined as polymers that undergo transformations in the biological environment by, for example, phagocytosis, through cellular activity [12, 13]. The bioabsorbable materials that are commonly used in cancer therapy are polyesters, polyanhydrides, polyphosphoesters, polysaccharides (e.g., chitosan, dextran, hyaluronic acid), and proteins (e.g., albumin, gelatin).

### Bioabsorbable synthetic polymers

**Fig. 1:**
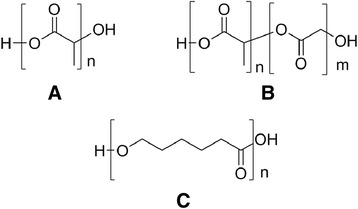
Structures of the aliphatic polyesters: **a** PLA, **b** PLGA, and **c** PCL

The aliphatic polyester poly(lactic acid) (PLA), the copolymer poly(lactic-co-glycolic acid) (PLGA), and poly(ɛ-caprolactone) (PCL) (Fig. 1) are by far the most used bioabsorbable synthetic polymers in the biomedical field [9, 12].

PLA can be obtained either from the polycondensation of lactic acid (LA) or by the ring opening polymerization (ROP) of lactide. This polyester can exist in four different morphological forms: poly(L-lactic acid) (PLLA), poly(D-lactic acid) (PDLA), the racemic poly(D,L-lactic acid) (PDLLA), and meso-PLA. PLLA and PDLA are semicrystalline, and PDLLA is amorphous in nature. Typically, these polyesters degrade by the hydrolysis of the ester linkage, giving LA as the degradation product [9, 14, 15]. In biomedical applications, only PLLA and PDLLA have been extensively studied. PDLLA presents a faster degradation time than PLLA, being more suitable for drug delivery applications [9].

PLGA is obtained through the copolymerization of LA (or lactide) and glycolic acid (GA) (or glycolide), and the properties (physical and biodegradation) of the copolyester can be easily tailored by changing the relative amounts of monomers in the final copolymer. The products of degradation are existing metabolites (LA and GA) of the human body [12, 16].

PCL is synthesized by the ROP of ɛ-caprolactone (ɛ-CL). It is a semicrystalline polymer with a low melting point range (59–64 °C), being suitable for the preparation of blends with other polymers or ceramic materials. Since PCL degrades slowly, it is mainly used in DDS that require long release profiles (e.g., more than 1 year). Nevertheless, if the ɛ-CL is copolymerized with LA or GA, a material that degrades faster is obtained. Upon degradation, PCL releases products with a less acidic character when compared to those released by PLA and PLGA [17].

**Fig. 2:**
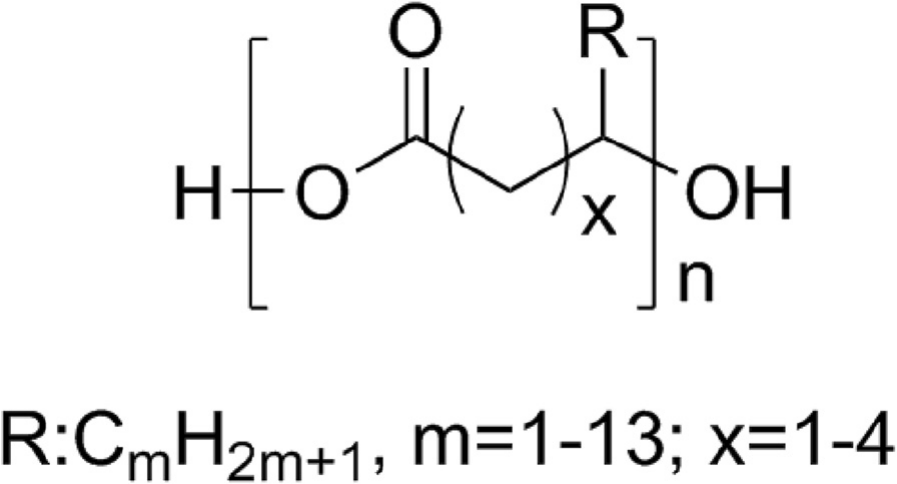
General structure of PHAs

Poly(hydroxyalkanoates) (PHAs) are aliphatic polyesters synthesized by microorganisms from various substrates used as carbon sources, under conditions of limiting nutrients [18]. Figure 2 shows the general structure of PHAs.

Until today, more than 150 different types of PHAs have been identified. These polyesters can be classified according to the number of carbons in the repeating unit in short chain length PHAs (3–5 carbon atoms) or in medium chain length PHAs (6–14 carbon atoms) [18, 19]. In the biomedical field, poly(3-hydroxybutyrate) (PHB) and the copolymers poly(3-hydroxybutyrate-*co*-4-hydroxybutyrate) (PHB4HB) and poly(3-hydroxybutyrate-*co*-3-hydroxyhexanoate) (PHBHHx) have attracted particular attention due to their excellent biocompatibility. PHB degrades slowly in vivo giving as degradation product D-3-hydroxybutyric acid, which is a component of blood plasma, serving as an energy reserve of extra-hepatic tissues during fasting, being well tolerated in vivo [20]. PHBHHx is very biocompatible in vivo since its degradation by-products, oligo(3-hydroxybutyrate) (OHB) and oligo(3-hydroxybutyrate-*co*-3-hydroxyhexanoate) (OHBHHx), present positive effects on cell growth [21, 22].

**Fig. 3:**
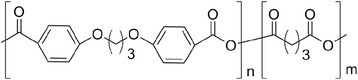
Chemical structure of poly[(1,3-bis-carboxyphenoxypropane)-co-(sebacic anhydride)]

Polyanhydrides are a class of surface-eroding polymers, and their hydrolysis rate can be controlled by the nature of the monomers (aliphatic or aromatic) used in the synthesis; aliphatic polyanhydrides degrade in days, whereas their aromatic counterparts degrade slowly over years. Thus, the careful selection of the monomers in the synthesis allows the preparation of polyanhydrides with a wide range of erosion rates. This class of polymers can be synthesized by different methods (melt polycondensation, ROP, interfacial polycondensation), but only the melt polycondensation allows the synthesis of these polymers with high molecular weight [23]. Polyanhydrides have been used as DDS, but attention should be paid regarding the drug that is going to be encapsulated to avoid undesirable reactions with drugs containing free amino groups or other nucleophilic groups, especially if the encapsulation occurs at high temperatures [12]. The most extensively studied polyanhydride copolymer is poly[(1,3-bis-carboxyphenoxypropane)-co-(sebacic anhydride)] (Fig. 3). This copolymer is approved by the Food and Drug Administration (FDA) for use in a biomedical device for the treatment of brain cancer that is marketed under the name Gliadel® Wafer [24].

**Fig. 4:**
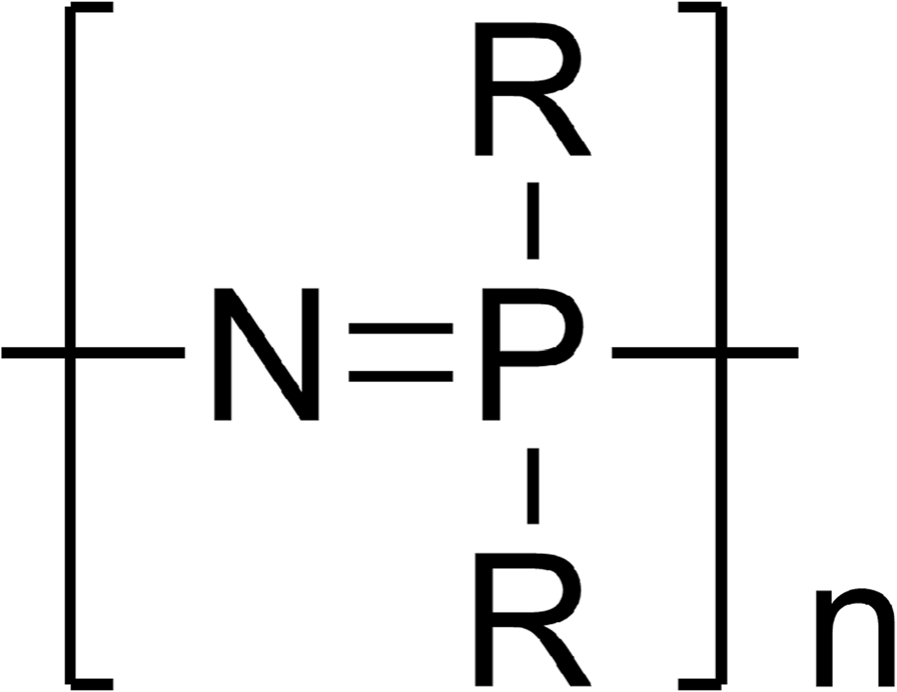
Generic structure of a polyphosphoester

Polyphosphoesters are an additional class of bioabsorbable polymers containing phosphorous (Fig. 4), developed in the 1970s by Lapienis and Penczek [25].

This class of polymers, due to the presence of the phosphoester bonds in their structure, is highly biocompatible. Depending on the type of functional group linked to the phosphorous atom, polyphosphoesters can be divided into polyphosphites (R_1_ = H), polyphosphonates (R_1_ = alkyl/aryl group), and polyphosphates (R_1_ = aryloxy/alkyloxy group) [26, 27]. The choice of the R_1_ and R groups allows the change of the polymers’ physico-chemical properties. Various synthetic routes including ROP, polycondensation, and transesterification are used to synthesize polyphosphoesters [27]. The degradation of polyphosphoesters occurs by hydrolytic and enzymatic cleavage of the phosphorous-oxygen bonds, giving phosphates, alcohols, and diols as degradation by-products. Due to the pentavalency of the phosphorous atom, the polyphosphazenes are able to chemically bind drugs or proteins. This is an interesting feature presented by this family of polymers since it enables the preparation of new polymer pro-drugs, with good in vivo compatibility [10]. Polyphosphoester-based microspheres have been used as DDS for paclitaxel (PTX) and are commercialized under the name Paclimer® [24].

### Natural polymers

Natural polymers can be divided into two main classes: proteins and polysaccharides. From a structural point of view, proteins are high molecular weight molecules in which α-amino acid residues are linked together by amide linkages and usually present a tridimensional folded structure [7].

Albumin and gelatin are the proteins with more relevance for the preparation of DDS for cancer therapy. Albumin is the most abundant protein in the human blood plasma, accounting for 50 % of its total mass. It presents a molecular weight of about 66.5 kDa and a diameter of 7.2 nm. Human serum albumin (HSA) has a multitude of functions in the human body [10, 11]. Among those, it is possible to mention the following: solubilization of long chain fatty acids, binder for bilirubin (resulting from heme breakdown), transport of metal ions (copper(II), nickel(II), calcium(II), and zinc (II)) in the blood stream, and major responsibility for colloid osmotic pressure of the blood, and upon hydrolytic breakdown, the resulting α-amino acids provide nutrition to the nearby tissues [28]. Albumin is a hydrosoluble protein, is stable in a pH range of 4 to 9, and can be heated at 60 °C for up to 10 h without deleterious effects. Since this protein is biodegradable, non-toxic, and non-immunogenic, it can be seen as an ideal candidate for DDS [10, 11, 28]. In the development of DDS for cancer therapy, the use of albumin can bring some advantages since this protein is used by the proliferating tumor cells for their nutrition, being readily taken up by a fluid phase endocytosis, followed by lysosomal breakdown. The α-amino acids resulting from the lysosomal digestion are used by the cancer cells as a source of nitrogen and energy [29]. In the market, there is already an injectable formulation of PTX-albumin nanoparticles, known as Abraxane®, that has been used in the treatment of breast cancer [28].

**Fig. 5:**
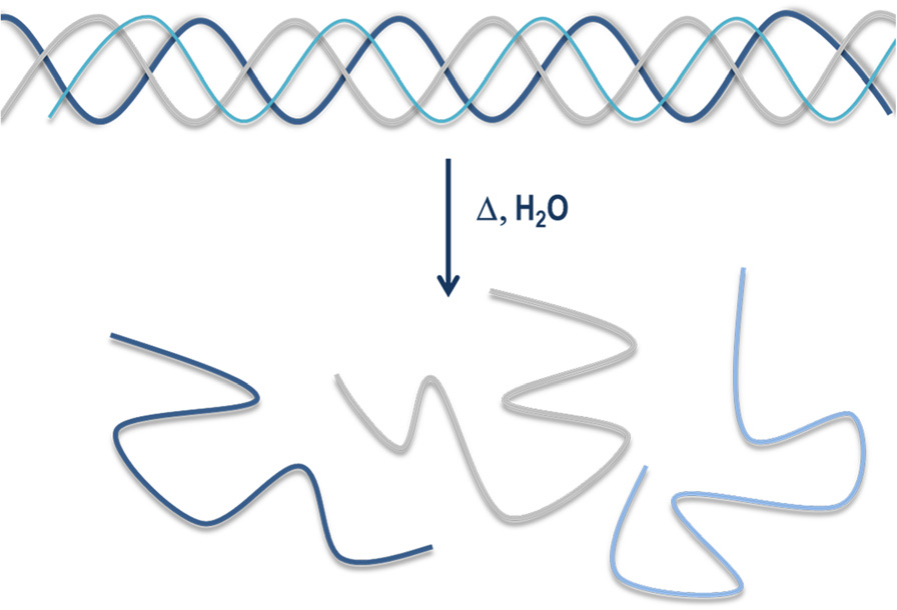
Schematic representation of the thermal denaturation of collagen (adapted from [7])

Gelatin is a protein obtained by the thermal denaturation of animal collagen, in which the triple helix of collagen is broken giving water-soluble strains (Fig. 5) [30, 31].

**Fig. 6:**
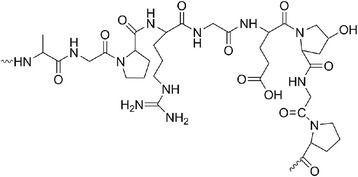
Structure of gelatin

If collagen is subjected to an acidic treatment before denaturation, type A gelatin, with an isoelectric point (IP) of 5, is obtained. In turn, if an alkaline treatment is used, type B gelatin, with an IP of 9, is obtained [31]. Gelatin is a polyampholyte whose structure comprises cationic (ca. 13 % in the total structure) and anionic groups (ca. 12 % in the total structure), along with the hydrophobic groups (ca. 11 % of the total structure). The positively charged groups are due to the presence of lysine and arginine residues, the negatively charged groups came from glutamic and aspartic acid, and the hydrophobic chain comprises residues of leucine, isoleucine, methionine, and valine. Glycine, proline, and hydroxyproline form the remaining part of the protein chain (Fig. 6) [30].

Gelatin undergoes a sol-gel transition in aqueous solution when the temperature is lowered below 35 °C. Because of its biodegradability and biocompatibility, gelatin has been used in the development of DDS for different biomedical applications, namely cancer therapy [30, 32].

**Fig. 7:**
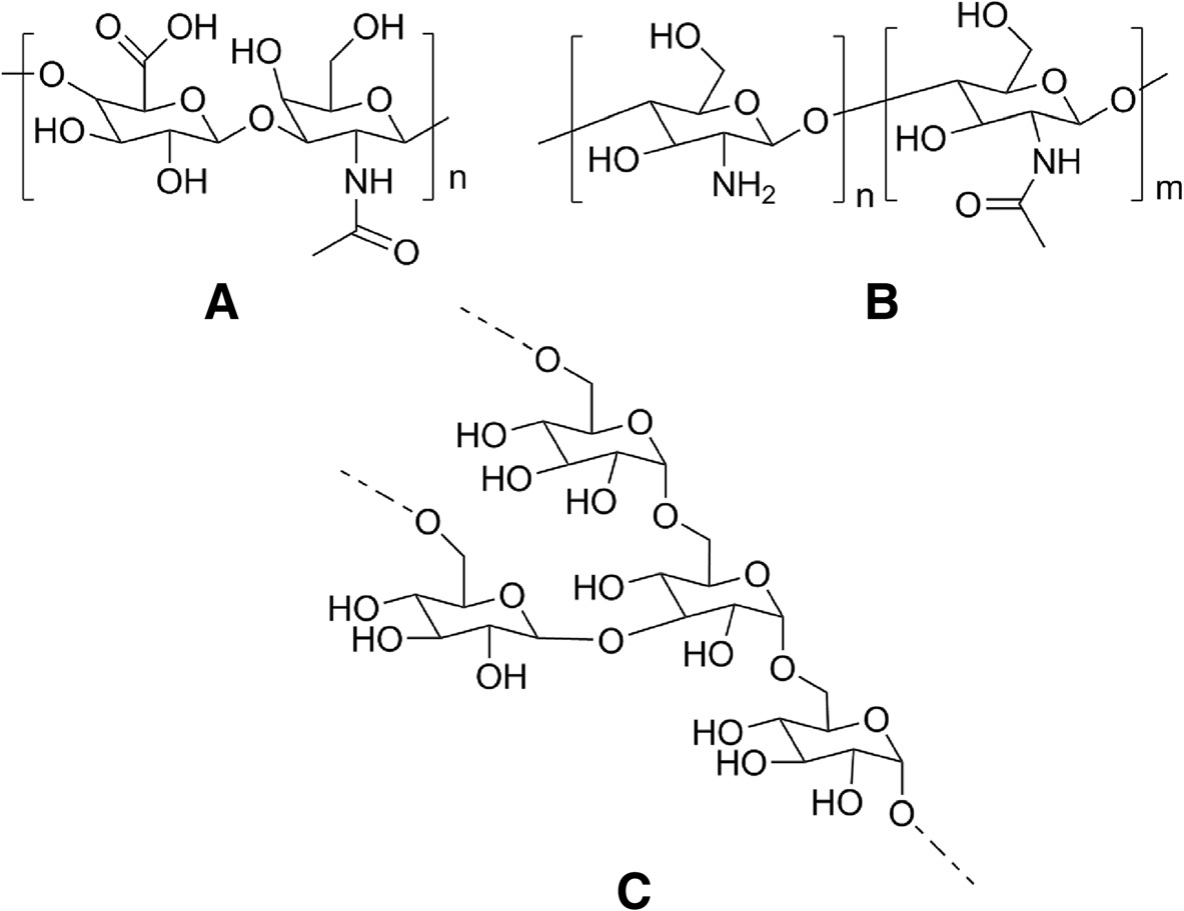
Structures of **a** hyaluronic acid, **b** chitosan, and **c** dextran

Polysaccharides are high molecular weight compounds, composed by monosaccharide repeating units. The monosaccharide units are linked together by *O*-glycosidic bonds that can occur on any hydroxyl group of the monosaccharide, yielding linear and branched structures. These biopolymers can have different origins, namely algal origin (e.g., alginate and carrageenan), plant origin (e.g., cellulose, pectin, and guar gum), and animal origin (e.g., chitosan, hyaluronic acid, chondroitin, and heparin) [33]. The hydrophobicity, solubility, and physico-chemical properties of the polysaccharides can be easily modified by different reactions, namely oxidation, sulfation, esterification, amidation, or by grafting methods [34]. In the context of cancer therapy, hyaluronic acid, chitosan, and dextran (Fig. 7) are the most explored polysaccharides.

Hyaluronic acid is a linear polysaccharide that belongs to the glycosaminoglycan family and is composed of alternating disaccharide units of *β*-1,3-*N*-acetyl-*D*-glucosamine and *α*-1,4-*D*-glucuronic acid connected by *β*(1 → 3) linkages. Its molecular weight can reach a few million Da [9, 10, 35]. This polysaccharide is the most abundant macromolecule in the intercellular matrix of conjunctive tissues such as vitreous humor, cartilage, umbilical cord, and synovial fluid [35, 36]. Regarding the applicability of hyaluronic acid in biomedical applications, several advantages can be pointed out: water-solubility, biodegradability, biocompatibility, non-toxicity, non-immunogenicity, and ease of chemical modification [33]. Another important feature of hyaluronic acid is its binding capacity to CD144 and CD168 (also known as receptor for hyaluronan-mediated motility, RHAMM) receptors, making it suitable to target cells that overexpress these receptors as cancer cells (squamous cell carcinoma, ovarian, colon, stomach, glioma, and many types of leukemia, lymphoma, and multiple myeloma) [33, 37–39].

Chitosan is a cationic polysaccharide obtained from the alkaline deacetylation of chitin, a polysaccharide widely found in Nature. It is composed by units of *β*(1 → 4)-2-amido-2-deoxy-D-glucan (*D*-glucosamine) and *β*(1 → 4)-acetoamido-2-deoxy-D-glucan (*N*-acetyl glucosamine) [33, 35]. The ratio between the *D*-glucosamine and *N*-acetyl glucosamine in the chitosan chain represents the deacetylation degree and usually presents values between 70 and 90 %. The deacetylation degree is a very important parameter since it controls the solubility, hydrophobicity, and the chitosan ability to interact with polyanions [40–42]. Chitosan is insoluble in water and in organic solvents but can be dissolved in slightly acidic aqueous solutions. The insolubility of chitosan is attributed to its rigid and highly crystalline structure due to the hydrogen bonds established between the primary amino groups and the acetoamido groups. A possible strategy to overcome this situation is the functionalization of the primary amino groups with other molecules (e.g., phthalic anhydride) in order to disrupt the crystalline structure [40]. Nevertheless, chitosan’s biocompatibility and biodegradability, along with its mucoadhesive properties, make it an interesting and widely used polymer for DDS [33, 35]. Another interesting and important feature is that chitosan revealed per se anti-cancer properties both in vitro and in in vivo [43].

Dextran is a water-soluble polysaccharide composed by α-1,6-linked D-glucopyranose units with branches extending mainly from the α-1,3- and occasionally from the α-1,2- and α-1,4- positions. It is produced by different bacteria from sucrose through the action of the enzyme dextransucrase. Dextran can present different molecular weights, molecular weight distributions, and degrees of branching depending on the conditions and bacterial strain used in its production [44, 45]. Dextran is easily biodegraded by the action of natural enzymes like dextran-(1,6)-glucosidase and dextranase. Additionally, it also shows good biocompatibility, non-immunogenicity, and non-antigenicity. Other important characteristics of dextran are related with its non-cell-binding ability and the capacity to resist protein adsorption, making it useful for intravenous administration [39, 44].

### Polymer-based DDS for anti-cancer therapy

#### Nanocarriers

Nanoparticles and micelles are undoubtedly the most used nanocarriers for anti-cancer therapy, and to maximize their utility and efficacy, they should obey a set of requisites. Thus, to be effective in cancer therapy, the nanocarriers should (i) increase the drug concentration in the tumor by passive or active targeting, (ii) decrease drug concentration in healthy tissues, (iii) improve the drug solubility to allow intravenous administration, (iv) release a minimum of drug during transit, (v) enhance the drug targeting specificity, (vi) improve internalization and intracellular delivery, (vii) protect the active compound against biochemical degradation, and (viii) be biocompatible [46].

Other important characteristics of nanocarriers that affect the drug release behavior are the size, composition, shape, surface charge and roughness, and the hydrophobic or hydrophilic nature [6]. Regarding the size, it is commonly accepted that the nanocarriers should not exceed the 400 nm to escape from the mononuclear phagocyte system (MPS) and be able to achieve extravasation into tumors by the enhanced permeability and retention (EPR) effect, which is more effective for nanocarriers with diameters below 200 nm [4]. Concerning the surface of the DDS, it must be hydrophilic and neutral to avoid plasma protein (opsonins) adsorption, which will delay the attack by the reticuloendothelial system (RES), increasing the blood circulation time.

#### Nanoparticles

Nanoparticles can be divided into two categories: nanospheres (matrix system) and nanocapsules (reservoir system). Nanospheres correspond to a solid polymer matrix; in this case, the active compound can be either molecularly dissolved or heterogeneously dispersed within the matrix. Nanocapsules are composed by a core and a shell; typically, the drug is in a cavity that is surrounded by the polymer shell [47]. Along the years, polymeric nanoparticles have proved to be efficient carriers for the sustained and prolonged release of anti-cancer drugs, mainly because the drug release behavior from the nanoparticles can be easily modulated by the type of polymer used [48]. The nanoparticles can be prepared by different techniques, namely emulsification, coacervation, nanoprecipitation, salting-out, dialysis, and electrospray [7].

Among the bioabsorbable synthetic polymers used to prepare nanoparticles for cancer treatment, PLGA is by far the most used. In 2011, Acharya and Sahoo published an excellent review about the use of PLGA nanoparticles in cancer therapy [49]. In 2012, Danhier et al. [50] devoted part of their review article entitled “PLGA-based nanoparticles: An overview of biomedical applications” to the use of PLGA nanoparticles in cancer treatment. Along the last years, PLGA-based DDSs have been developed and tested as carriers of different drugs to treat different types of cancer, viz., pancreatic [51], osteosarcoma [52], breast [53–55], lung [56], and prostate [57].

Zhou et al. [58] prepared PLGA nanoparticles for the delivery of small interfering ribonucleic acid (siRNA) in tumors. By sequential steps, the authors managed to give the nanoparticles a set of controllable synergistic functions, namely (i) stabilization and controlled release of siRNA; (ii) encapsulation of siRNA for gene knockdown; and enhancement (iii) of endosomal escape, (iv) of siRNA potency, and (v) of circulatory time, (vi) cell penetration, and (vii) tumor targeting. The in vivo results showed that the PLGA-based DDS was effective in controlling the growth of tumor and in prolonging the knockdown of the PLK1 gene, which is essential for mitosis. This gene is overexpressed in many types of cancer, being associated with tumorigenesis. More recently, Wang et al. [59] co-encapsulated doxorubicin (DOX) and epidermal growth factor receptor (EGFR) siRNA in PLGA nanoparticles, in which the angiopep-2 (ANG) was conjugated. ANG is a brain-targeted peptide, and its use could bring advantages in overpassing the blood brain barrier (BBB) that is known to restrict the access of drugs to the brain. The DDS was tested in vitro making use of a U87MG cell line (human primary glioblastoma cell line). It was found that it was able to efficiently release the DOX and siRNA, contributing to an inhibition of the cell growth, apoptosis, and EGFR silencing. The in vivo tests showed that DDS was capable of crossing the BBB, and the therapy using both EGFR siRNA and DOX contributed to extend the survival time of the U87MG-glioma-bearing mice.

Schleich et al. [60] developed theranostic PLGA nanoparticles loaded with PTX and superparamagnetic iron oxide (SPIO) (a contrast agent for magnetic resonance imaging), using the emulsion-diffusion-evaporation method. The nanoparticles were spherical in shape with a diameter of 240 nm. The in vivo anti-tumor efficacy was tested with CT26 (colon carcinoma cells)-tumor-bearing mice, and the results showed that the loaded nanoparticles were effectively uptaken by the CT26 cells and were able to delay the regrowth of the CT26 tumor.

Shi et al. [61] studied the effect of conjugating vascular endothelial growth factor receptor (VEGFR) to the surface of PLGA nanoparticles encapsulating PTX. VEGFR can be considered as an ideal targeting moiety in cancer treatment, since it is overexpressed on the surface of a variety of tumor cells and plays a key role in the mitosis and angiogenesis processes. The PLGA nanoparticles were prepared by the emulsion-solvent evaporation method, and then, the VEGFR was conjugated to the nanoparticles’ surface. The results showed that 16.6 wt.% of VEGFR was conjugated to the nanoparticles’ surface. The diameters of the particles ranged from 390 nm (for blank nanoparticles) to 710 nm (for particles containing VEGFR and PTX). The anti-tumor activity of PTX encapsulated in the VEGFR-PLGA nanoparticles was studied in vitro making use of 7721 human hepatocarcinoma cells and A549 human lung cancer cells. The results showed that the PTX-loaded VEGFR-PLGA nanoparticles have a high inhibitory activity of tumor growth when compared with native PTX or with PTX-loaded PLGA nanoparticles.

Very recently, Iodice et al. [62] encapsulated gold nanoparticles (AuNPs; 6 nm) in PLGA nanoparticles coated with a lipid poly(ethylene glycol) (PEG) monolayer and used the ensuing nanostructures in photothermal therapy. The nanostructures presented diameters ranging from 100 to 180 nm, being the size dependent on the amount of gold. The cytotoxic effect of these nanostructures was tested in 2D monolayers of breast cancer cells (SUM-159) and 3D tumor spheroids of glioblastoma multiform cells (U87MG). The overall results suggest that the encapsulation of the AuNPs in the coated PLGA nanoparticles improved the photothermal ablation.

Although less used than PLGA, PLA-based nanoparticles also showed to be promising as anti-cancer drug DDS.

**Fig. 8:**
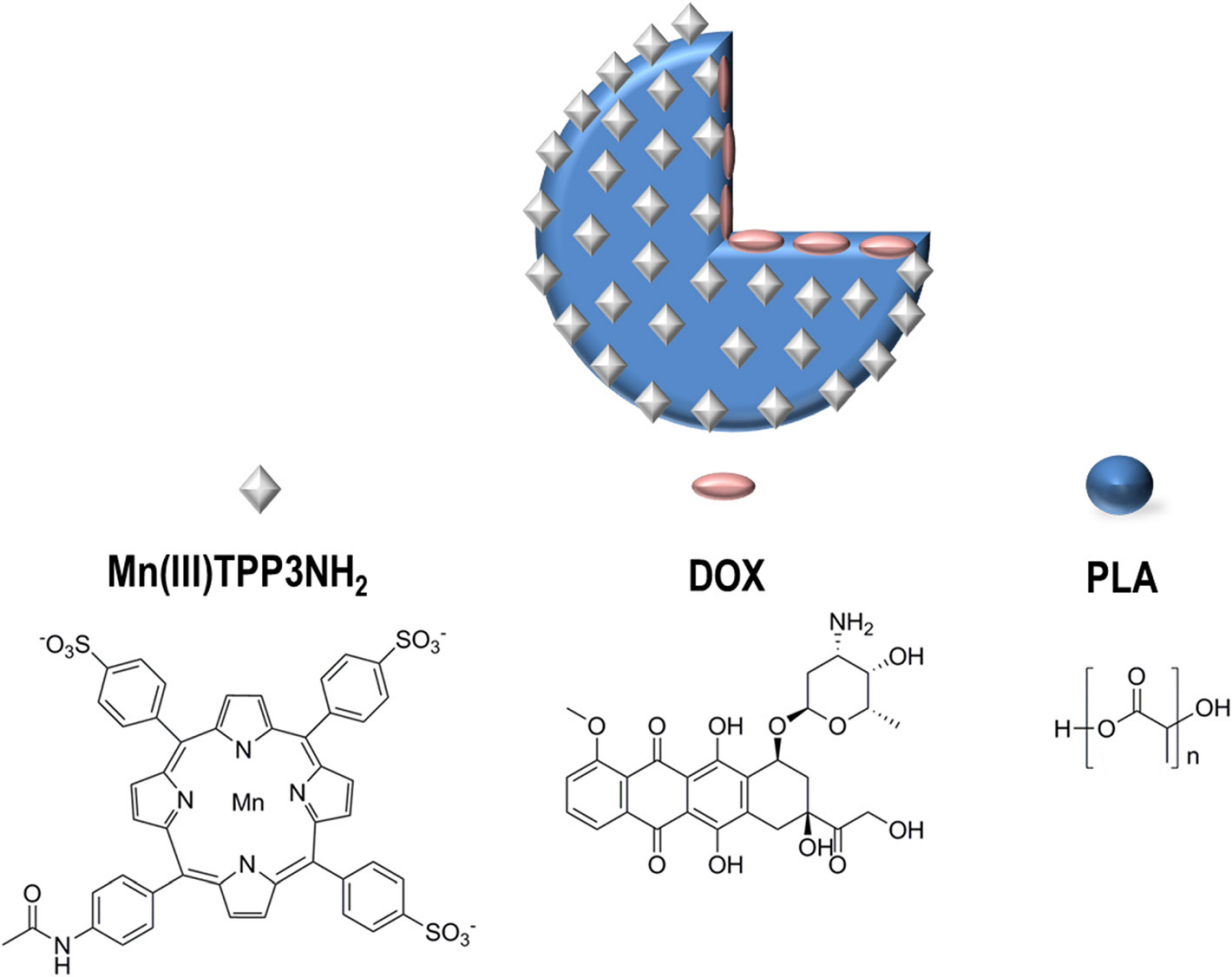
Representation of the PLA nanoparticle developed by Jing et al. (adapted from [63])

Jing et al. [63] prepared theranostic DOX-loaded PLA nanoparticles (Fig. 8) by a nanoemulsion method, followed by the conjugation of a Mn-porphyrin on the surface of the nanoparticles.

The in vitro release studies showed that the nanoparticles are sensitive to pH; at neutral pH, the DOX release was slow, while in acidic medium, the release was significantly faster. The results also demonstrated that the nanoparticles are effective in inhibiting the growth of HeLa and HT-29 cells. Additionally, the nanoparticles were subjected to a magnetic resonance imaging (MRI) scanning analysis, and it was shown that the nanoparticles had higher longitudinal relaxivity in water than the Mn-porphyrin, making them excellent candidates to be used in T_1_-weighed MRI. Also in the field of theranostic PLA nanoparticles, Du et al. [64] reported the preparation of PLA nanocarriers to release endostar, a recombinant human statin that has been shown to inhibit tumor angiogenesis. The surface of the nanoparticles was conjugated with a peptide, GX1, which is a tumor vasculature endothelium-specific ligand that holds great potential as a targeted vector and anti-angiogenic agent. Additionally, the nanoparticles were labeled with the near-infrared dye IRDye 800CW to allow the monitorization of the biodistribution and tumor-targeting efficacy of nanoparticles by means of fluorescence molecular imaging. Colorectal cancer was used as the model disease. The in vivo tests performed on colorectal-tumor-bearing mice showed that the endostar-loaded nanoparticles were more effective in treating the tumor than the native endostar. The presence of the dye was useful to follow the biodistribution of the nanoparticles in vivo.

Very recently, Yang et al. [65] developed a DDS based on PLA nanoparticles for the treatment of lung cancer metastasis. The nanoparticles encapsulating docetaxel (DTX) were prepared by the single emulsion method, and a targeting peptide was conjugated on their surface. The targeting peptide, screened from lung carcinoma stem cells, showed a high specific-binding ability to pulmonary adenocarcinoma tissue. The anti-metastatic efficacy of the nanoparticles was tested in vivo making use of a nude mice model of liver metastasis. The results revealed that the peptide had a key role in the anti-metastatic efficacy of the nanoparticles.

Natural polymers due to their inherent advantages are also interesting candidates to prepare nanoparticles for anti-cancer drug delivery [39]. In recent years, albumin-based nanoparticles have been prepared and tested as DDS in different types of cancer. Ji et al. [66] prepared albumin nanoparticles decorated with arginylglycylaspartic acid (RGD) to be used as DDS in the treatment of pancreas cancer. RGD was bound to the nanoparticles’ surface with the aim of targeting integrin αvβ3, which is expressed in pancreatic tumor cells. The nanoparticles were prepared by the desolvation-crosslinking method, conjugated with RGD and loaded with fluorescein isothiocyanate (FITC), in order to trace their biodistribution. Gemcitabine was used as the anti-cancer drug. The in vitro tests, conducted on BxPC-3 cells, showed that the presence of RGD at the surface of the nanoparticles led to a higher intracellular uptake when compared with the nanoparticles without the tripeptide. The nanoparticles, loaded with gemcitabine, have shown improved anti-tumor efficacy, both in vitro and in vivo.

Recently, Bhushan et al. [67] reported the encapsulation of niclosamide in albumin nanoparticles, using the desolvation method, and tested the efficacy of the resulting DDS in breast cancer and lung cancer cell lines. The in vitro tests showed that the nanocarrier improved significantly the tumor inhibition capacity of the drug by inducing a significant amount of apoptosis in the treated cancer cells.

In a very interesting contribution, Mocan et al. [68] encapsulated AuNPs in albumin carriers and used the ensuing system in the photothermal treatment of liver cancer. The results indicate that the nanoparticles were selectively internalized by the HepG2 cells, meaning this internalization was mediated by the gp60 receptors’ targeting. It was also shown that after irradiation, the photoexcitation of the nanoparticles exhibited a higher apoptosis capacity.

Gelatin is another protein that has been formulated as nanoparticles for cancer therapy [30]. Lee et al. [69] prepared a tumor-targeted siRNA delivery system making use of this natural polymer. Since gelatin has a low binding affinity to siRNA, the authors functionalized both the siRNA (in the form of a polymer, poly-siRNA) and gelatin with thiol groups. Under the optimized conditions, the siRNA was covalently linked to gelatin, by disulfide bonds, and the nanoparticles were formed. The results showed that nanoparticles were effective in protecting the siRNA from enzymatic degradation and were able to release siRNA when exposed to reductive environments. The efficacy of the nanoparticles as DDS for cancer therapy was tested in red fluorescence protein (RFP)-expressing melanoma cells. It was found the delivery system was efficient in down-regulating the targeted gene expression. The in vivo tests showed that the tumor accumulation was more effective for the nanoparticles than for the naked poly-siRNA. Additionally, the nanoparticles induced tumor RFP gene silencing in vivo, without inducing significant toxicity.

Xu et al. [70] investigated the in vivo biodistribution and targeting potential of three groups of type B gelatin nanoparticles (thiolated nanoparticles, thiolated nanoparticles modified with PEG, and thiolated nanoparticles modified with PEG bearing a EGFR-targeting peptide), in human pancreatic carcinoma (Panc-1)-bearing SCID Beige mice. The results revealed that EGFR-targeted nanoparticles show an enhancement of the targeting efficiency. Driven by the excellent results of biodistribution and accumulation in tumors, the same authors used these particles as DDS for gemcitabine, a widely used chemotherapeutic for the treatment of pancreas cancer [71]. Long et al. [72] encapsulated DOX in thiolated gelatin nanoparticles bearing the EGFR-targeting peptide and studied their potential as DDS for lung cancer. The in vitro tests conducted on EGFR overexpressing A549 and H226 lung cancer cells revealed that the nanoparticles were internalized by the cells via a receptor-mediated endocytosis. The inhibition of the tumor growth was dependent on the dose of nanoparticles used. The in vivo anti-tumor efficacy was evaluated in A549-tumor-cell-bearing nude mice using inhalation as the administration method. The results showed that the EGFR-bearing nanoparticles allowed maintaining the DOX concentration in the lungs at a high level, even after 24 h of administration. Moreover, the nanoparticles cause a high percentage of tumor inhibition (90 %), when compared to native DOX. A mice survival percentage of 100 %, during the 2 weeks of the tests, was also observed.

Among the polysaccharides, chitosan is, by far, the most used to formulate nanoparticles for cancer therapy [73]. This fact can be related with its anti-cancer properties, already proved both in in vitro and in in vivo studies [43, 74]. In 2015, two reviews about the use of chitosan nanoparticles in tumor-targeted drug delivery were published [75, 76].

Dextran is another polysaccharide used in the preparation of nanoparticles or as a coating for magnetic nanoparticles for anti-cancer DDS purposes. Sagnella et al. [77] prepared dextran nanoparticles bearing aldehyde functionalities that were able to form an acid labile linkage with DOX. The anti-cancer efficacy of the developed DDS was tested in vitro making use of both (2D) SK-N-BE(2) monolayers and (3D) SK-N-BE(2) tumor spheroids. The results show that the nanoparticles were rapidly and effectively internalized by the (2D) SK-N-BE(2) monolayers. Regarding the tumor spheroids, the results indicate that the nanoparticles were able to penetrate deeper in the solid tumor when compared with DOX alone. Very recently, dextran nanoparticles were used to encapsulate micro ribonucleic acids (miRNAs) (miR-199a-3p and miR-let-7a) that can act as a chemotherapeutic agent. The miRNAs were encapsulated in lipid-modified dextran nanoparticles, and the anti-cancer efficacy of the DDS was tested with two different osteosarcoma lines, viz., KHOS and U-2OS. The results showed that the miRNAs were successfully delivered to the tumor cells and effectively inhibit the growth and proliferation of the osteosarcoma cells in vitro [78]. Peng et al. [79] used dextran-coated superparamagnetic iron nanoparticles (SPIONs) as DDS for DOX. The complex DOX-nanoparticles showed a pH-dependent drug release, being faster at pH values below the physiologic pH. The in vivo tests, carried out under an external magnetic field, performed on a rabbit VX2 liver tumor model showed that the nanoparticles were more effective in inhibiting the tumor growth and in increasing the animal survival rate than free DOX.

Another interesting strategy that has been used is the preparation of nanoparticles making use of both synthetic and natural polymers. For instance, Zhou et al. [80] prepared nanoparticles based on poly(ɛ-caprolactone) (PCL)-grafted galactosylated chitosan to be used as a carrier for curcumin. The nanoparticles were prepared through the ionotropic gelation method, using tripolyphosphate as the gelification agent. The encapsulation efficiency of the nanoparticles was between 25 and 80 %, with particle sizes ranging from 110 to 140 nm. The most promising result in terms of release behavior (sustained release) was observed for the copolymer 10 % galactosylated and with PCL weight percentages below 40 %. The nanoparticles were effectively uptaken by the human hepatocellular carcinoma (HepG2) cells, confirming the hepatocyte-targeting feature of the DDS. The nanoparticles showed best results in terms of inducing apoptosis and necrosis of the HepG2 cells than free curcumin.

#### Micelles

**Fig. 9:**
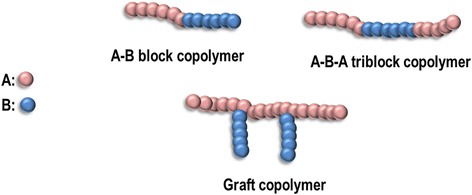
Different designs of the amphiphilic copolymers used in the preparation of micelles

Micelles are nanosized (10–100 nm) aggregates of amphiphilic copolymers, comprising a hydrophobic core, in which a poorly water-soluble drug can be loaded, and a hydrophilic shell (or corona), that allows the load of hydrophilic drugs and provides stability to the micelle [81, 82]. The micelles are obtained when the concentration of amphiphilic copolymer in aqueous medium reaches the critical micelle concentration (CMC). Above the CMC, the formation of the micelles is spontaneous and driven by the dehydration of the hydrophobic part of the polymer. In addition, the formation of van der Waals bonds allows the hydrophobic polymers to join and form the micelle core [83]. Typically, the amphiphilic blocks can have different designs: A-B (diblock copolymers), A-B-A (triblock copolymers), and grafted copolymers (Fig. 9).

The final characteristics of the micelles can be easily modulated by choosing the blocks that will comprise the block copolymer. The hydrophilic block, for instance, besides conferring stabilization to the micelles can also be used to increase the blood circulation time. PEG is by far the most used hydrophilic block in polymeric micelles, since it is highly hydrophilic, is an efficient structural stabilizer, and is biocompatible [84, 85].

There are several advantages of polymeric micelles that make them attractive nanocarriers for cancer therapy. Micelles can solve the solubility issues of a vast part of chemotherapeutics, which are hydrophobic in nature, because they can accommodate this type of drugs in their hydrophobic core. The size presented by the micelles is too large for extravasation from normal vessels walls and renal excretion but allow extravasation from tumor blood vessels. An enhanced EPR effect, which is crucial for passive targeting, is also observed when micelles are used. Additionally, micelles can change the drug internalization route decreasing the P-glycoprotein (P-gp) efflux effect, which acts as a mechanism of resistance of some types of cancers to treatment with some drugs [85, 86].

The interest and the importance of this type of carriers in the field of cancer treatment are exhibited on the seven formulations that are currently under clinical trials (Table 1).

**Table 1:** Polymeric micelles in clinical trials for cancer therapy [4, 83, 86–88]

Polymeric micelle	Block copolymer	Drug	Diameter (nm)	Therapeutic purpose	Clinical phase
Genexol-PM™	MonomethoxyPEG-*b*-PDLLA	PTX	20–50	Metastatic breast cancer	IV
				Advanced urothelial cancer	II
				Advanced head and neck cancer	II
				Advanced non-small-cell lung cancer	II
				Ovarian cancer	I
				Advanced or metastatic pancreatic cancer	II
Nanoxel-PM™	MethoxyPEG-*b*-PDLLA	DTX	25	N.A.	I
NK012	PEG-*b*-Poly(glutamic acid)	SN-38	20	Breast cancer	II
NK105	PEG-*b*-Poly(aspartic acid)	PTX	85	Advanced stomach cancer/breast cancer	III
NK911	PEG-*b*-Poly(aspartic acid)	DOX	40	Various solid tumors	II
NC-4016	PEG-*b*-Poly(glutamic acid)	Oxaliplatin	30	Various solid tumors	I
NC-6004	PEG-*b*-Poly(glutamic acid)	Cisplatin	30	Pancreatic cancer	III
NC-6300	PEG-*b*-Poly(aspartate-hydrazone)	Epirubicin	60	Various solid tumors	I

**Fig. 10:**
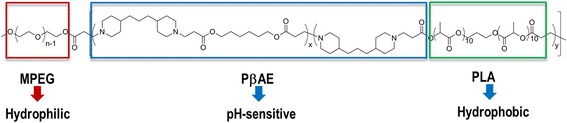
Structure of the MPEG-*b*-(PLA-co-PAE)

Besides the micelles that are currently under clinical trials, many others have been developed during the last years [89]. Micelles can have other “functions” beyond carrying the chemotherapeutic agent that could enhance their performance regarding anti-cancer activity. These carriers can be formulated to be stimuli-responsive [90–92] or to bear a targeting ligand at the surface, making them able to target a specific tumor [93–95]. Micelles can also be used for theranostic purposes as reported in the literature [96, 97]. Amphiphilic block copolymers based on PEG polyesters (PLA, PLGA, PCL) are clearly the most studied materials in the formulation of micelles [98]. However, other materials can be included; other materials in the block copolymer or other materials can be used to prepare polymeric micelles towards cancer therapy. In a very interesting contribution, Zhang et al. [99] prepared a pH-responsive triblock copolymer, viz., poly(ethylene glycol) methyl ether-*b*-(poly(lactic acid-*co*-poly(β-amino ester))) (MPEG-b-(PLA-co-PAE)) with different PLA/PAE ratios, and used it as a DDS for DOX (Fig. 10).

The CMC of these structures in aqueous solution ranged from 1.2 to 9.5 mg/mL, and the values have shown to be dependent on the amount of PLA in the copolymer, being lower for higher amounts of the polyester. The DOX loading was about 18 %, and the in vitro release results showed that the release of the drug was significantly accelerated when the pH decreased from 7.4 to 5. This result was attributed to a looser micellar structure due to the protonation of the PAE moieties. The in vitro cytotoxicity tests carried out on a HepG2 cellular line showed that the unloaded micelles do not induce cytotoxicity, contrarily to what was observed with the loaded DOX micelles.

Kim et al. [100] used micelles composed by poly(ethylene oxide)-*b*-poly(3-hydroxybutyrate)-*b*-poly(ethylene oxide) (PEO-PHB-PEO) triblock copolymers to encapsulate DOX. The incorporation of the drug in this copolymer is enhanced due to the hydrophobicity of the PHB central block that can lead to more stable micelles with high drug encapsulation efficiencies. The distribution and cytotoxicity of DOX-loaded PEO-PHB-PEO micelles was evaluated on a monolayer culture of SiHa human cervical carcinoma (HeLa) cells and on 3DSiHa multicellular spheroids (MCS). In order to understand the mechanism of micelles’ cell uptake and penetration, fluorescent-labeled PEO-PHB-PEO micelles were prepared and the in vivo anti-proliferative activity was studied in nude mice models. The results demonstrated that the micelles improved the efficiency of DOX penetration in 3D MCS cultures with effective cell killing, showing to be promising devices to deliver DOX for cancer therapy.

**Fig. 11:**
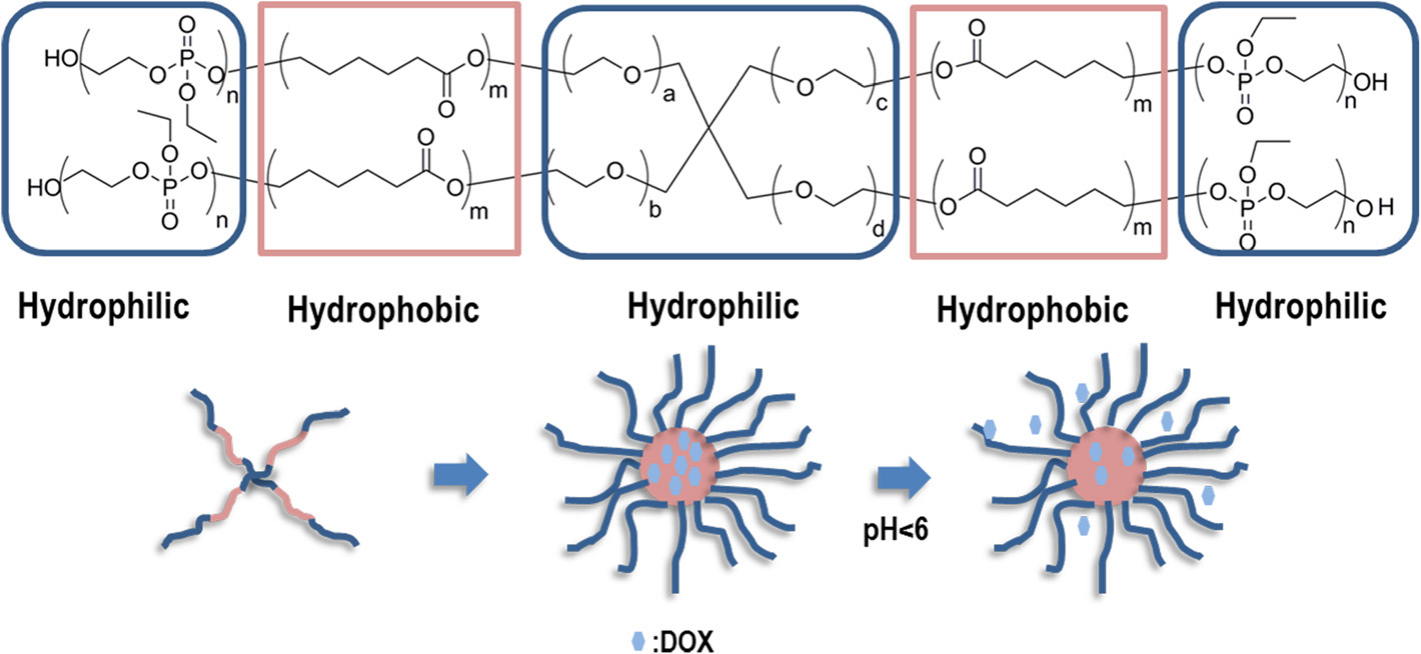
Structure of the PCL-PEEP star-shaped copolymer and micelles’ formation

Cuong et al. [101] synthesized a star-shaped PCL that was further extended with a terminal block of poly(ethyl ethylene phosphate) (PEEP) to yield a PCL-PEEP star-shaped copolymer. The copolymer showed the ability to form micelles in aqueous solutions and was used to encapsulate DOX (Fig. 11).

The in vitro release test showed that DOX was delivered in a pH-dependent manner, being faster at pH = 5.4 than at physiological pH. The DOX-loaded micelles presented enhanced cytotoxicity for both drug-sensitive and drug-resistant human breast cancer cell lines (MCF-7). The evaluation of the cellular uptake of the DOX-loaded micelles was done by means of confocal microscopy. A strong fluorescence in the cytoplasm of the cells indicates that the micelles were internalized through endocytosis. Interestingly, the drug-resistant cells showed higher cellular uptake than their drug-sensitive counterparts.

Transferrin (Tf)-conjugated polyphosphoester hybrid micelles loaded with PTX were prepared by Zhang et al. [102], and their in vitro and in vivo brain-targeting ability was studied. The micelles were formed from a blend of the copolymer PCL-co-PEEP with a maleimide-PCL-PEG copolymer (needed for the attachment of Tf) by the dialysis method. The micelles had diameters of *ca.* 87 nm, high drug entrapment efficiency (ca. 89.9 %), and caused negligible hemolysis. The release tests showed that PTX can be delivered in a sustained manner over a period of 72 h. The in vitro cellular uptake tests were carried out with brain microvascular endothelial cells (BMECs), since they closely represent the barrier property of BBB in vivo. BBB highly express the Tf receptor, and because of that, the presence of Tf could mediate the transport of micelles across the BBB. The results indicated that the cellular uptake by the Tf-conjugated micelles was almost twofold higher than that observed for the micelles without Tf. The anti-cancer activity of the Tf-conjugated micelles was evaluated in vivo using U87MG-glioma-bearing mice, and the results were very promising; the micelles effectively accumulated in the brain tumor and showed stronger anti-glioma capacity than Taxol®, a chemotherapeutic widely used in glioma treatment. Moreover, an increase of 6 days in the mean survival time of the mice was observed for the group treated with the Tf-conjugated micelles, when compared with Taxol®.

**Fig. 12:**
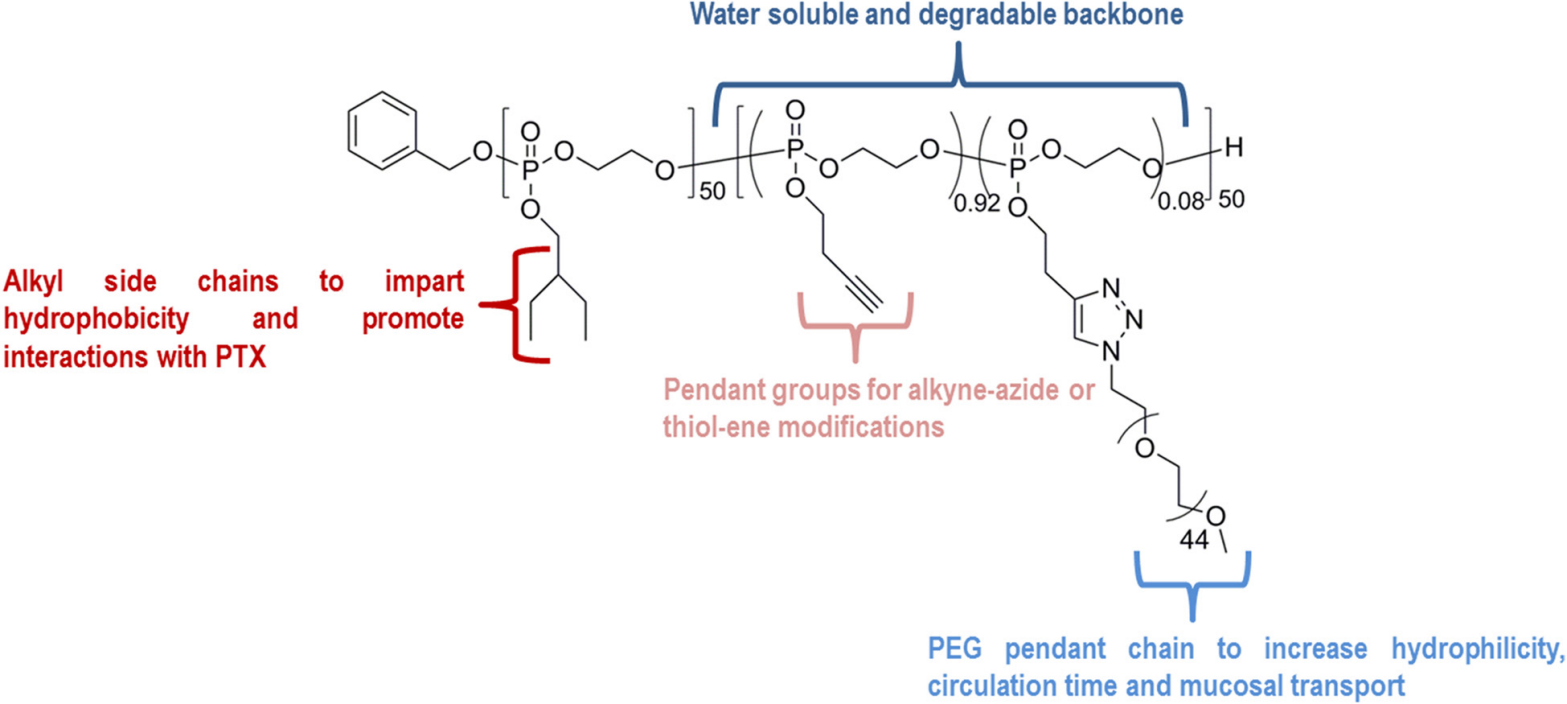
Structure of the PBEP-*b*-PBYP-*g*-PEG block terpolymer (adapted from [103])

Very recently, Zhang et al. [103] prepared an amphiphilic block terpolymer poly(2-ethylbutoxy phospholane)-*b*-poly(2-butynylphospholane)-*g*-poly(ethylene glycol) (PBEP-*b*-PBYP-*g*-PEG) (Fig. 12) and used it to formulate uncrosslinked and crosslinked micelles (shell crosslinked knedel (SCK)-like nanoparticles) for the encapsulation of PTX. The SCKs were obtained by the micelles’ shell crosslinking through a thiol-yne “click chemistry”.

The in vitro release tests showed that the SCKs provided a slower release PTX when compared with the uncrosslinked micelles. The anti-tumor efficacy of the micelles and SCKs were tested against osteosarcoma cell lines (CCH-OS-O and SJSA) that are known to metastatize in the lungs. The in vitro tests showed that the micelles and SCKs had similar cytotoxicities to the commercially available Taxol-mimicking formulation. The in vivo biodistribution and pharmacokinetics were studied after intratracheal delivery, and the results proved that the shell crosslinking has a key role in controlling the rate of extravasation from the lungs; SCKs were retained almost twice than their uncrosslinked counterparts.

Micelles prepared by the use of both synthetic and natural polymers are gaining importance. Dai et al. [104] prepared micelles from a cellulose-*g*-PLA copolymer by the nanoprecipitation method and used them to encapsulate betulinic acid (BA). The micelles were spherical in shape with sizes ranging from 100 to 170 nm. The in vitro release tests showed that the BA release depended on the amount of PLA in the copolymer; higher amounts of PLA led to a more sustained release, without a less accentuated initial burst. The in vitro cytotoxicity tests carried out on A549 and LLC cell lines showed that the bare micelles do not elicit any cytotoxic response, contrarily to what was observed with the BA-loaded micelles. Moreover, the BA-loaded micelles had a higher anti-cancer activity when compared with the free drug. The in vivo tests conducted on a mouse tumor xenograft model revealed that the micelles were more effective in promoting tumor inhibition than the BA itself.

Han et al. [105] prepared an amphiphilic copolymer based on gelatin, PLA, and a lipid moiety, 1,2-dipalmitoyl-sn-glycero-3-phosphoethanolamine (gelatin-co-PLA-DPPE). The micelles were prepared from this copolymer, by the nanoprecipitation and double emulsion methods, and used to encapsulate DOX (in its salt form). The release of DOX was studied, and the results showed that the drug release was faster for pH = 5 than for pH = 7.4. The pH-dependent release was attributed to the presence of the lipid in the copolymer structure. The in vitro cytotoxicity tests, performed on an A549 cell line, indicated that the unloaded micelles did not have any cytotoxic effect, contrarily to what was observed with the DOX-loaded micelles. The in vivo tests also suggested that the DOX-loaded micelles were effective in suppressing the tumor growth in mice.

**Fig. 13:**
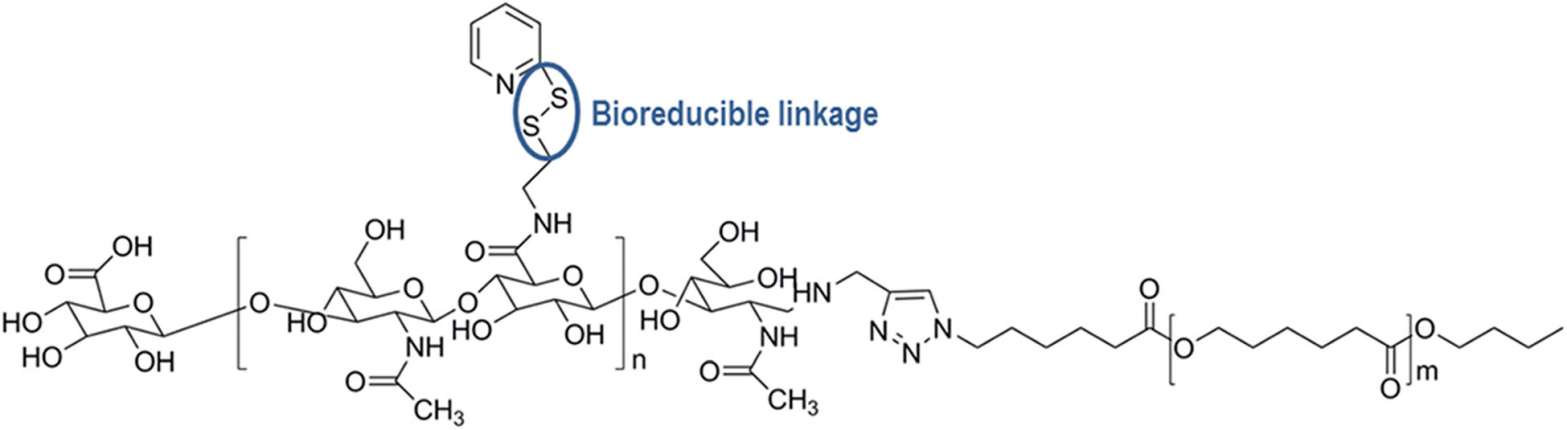
Structure of the amphiphilic hyaluronic acid-*b*-PCL copolymer

Huang et al. [106] prepared PCL-grafted hyaluronic acid nanoparticles for the oral delivery of anti-cancer drugs, namely PTX. The PTX-loaded PCL-grafted hyaluronic acid nanoparticles were prepared by a dialysis method, followed by coating with chitosan using the layer-by-layer technique mediated by electrostatic interactions. This DDS is sensitive to pH; in a 3 to 6.8 pH range the core-shell structure of the nanoparticle is maintained intact, but with an increase of the pH value to 7.4, the detachment of the chitosan layer is observed. The in vitro tests demonstrated that the PTX release is triggered by the presence of hyaluronidase-1. In the esophageal squamous carcinoma (EC109) cell line, the nanoparticles were effectively internalized via a receptor-mediated endocytosis and rarely uptaken by normal fibroblast (NIH3T3) cells. Han et al. [107] prepared disulfide crosslinked micelles based on the amphiphilic hyaluronic acid-*b*-PCL copolymer for DOX encapsulation (Fig. 13). The in vitro tests reveal that the crosslinking of the micelles’ shell slows the release of DOX in physiological pH, but in the presence of glutathione (GSH), a reductive agent found in the cells’ cytoplasm, the release was noticeably improved. Using a SCC7 cellular line, the in vitro studies showed that the nanoparticles were efficiently internalized by the cells and the DOX was successfully release at the intracellular level, by a mechanism based on their binding to the receptor CD44. The in vivo tests on SCC7-tumor-bearing mice demonstrated that the DOX-loaded crosslinked micelles were able to suppress the tumor growth.

Micelles based on methoxyPEG conjugated to denaturated bovine serum albumin were prepared and used as a carrier for camptothecin (CPT) by Zhang et al. [108]. The unloaded micelles presented sizes of 238 nm, whereas the diameters of their loaded counterparts were 150 nm. The authors attributed the small size of the loaded micelles to favorable hydrophobic interactions that led to more compact micelles. The CPT maximum loading was *ca.* 24 %. The cellular uptake tests carried out on HeLa cells showed that the micelles were easily uptaken by the cells, even more than the “unmodified” bovine serum albumin (BSA). Concerning the anti-cancer performance of the CPT-loaded carriers, it was found that they possess similar activity to that of free CPT.

**Fig. 14:**
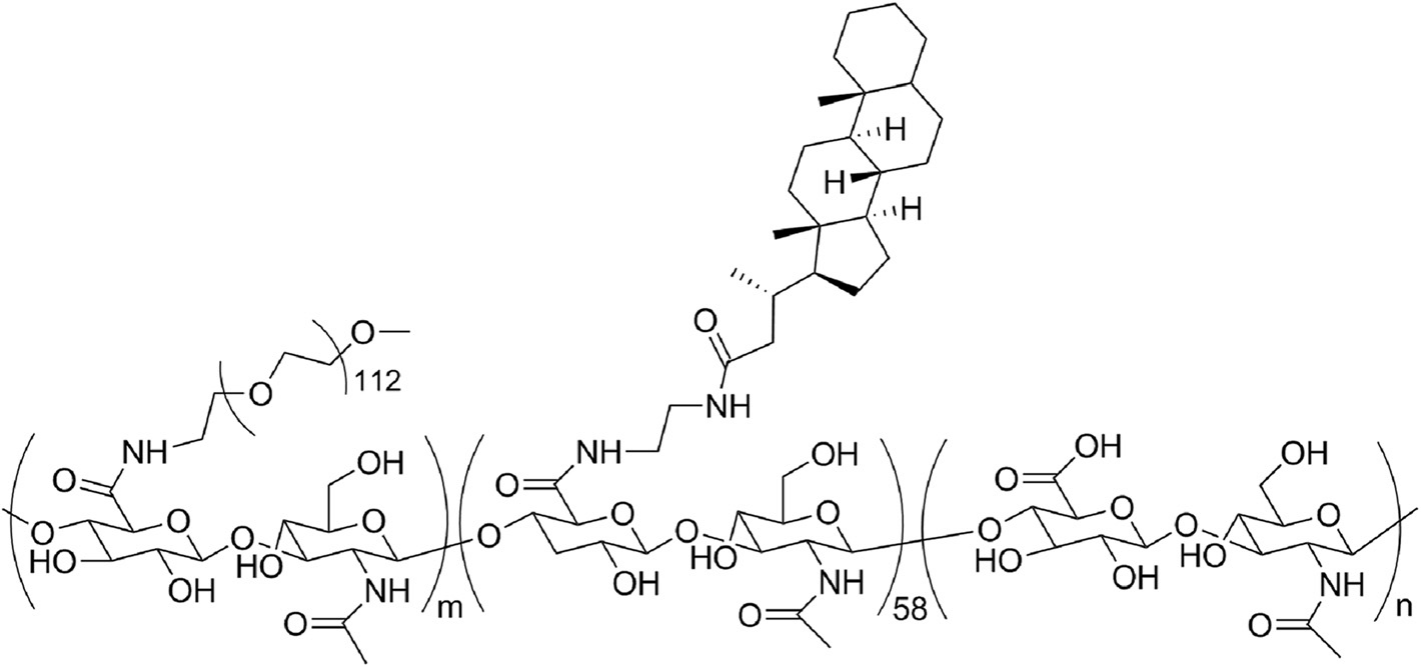
Structure of the PEG-conjugated hyaluronic acid

Choi et al. [109] prepared micelles based on PEG-conjugated hyaluronic acid (Fig. 14) and studied the effect of the presence of PEG in the biodistribution of the micelles.

The results suggested that the PEGylation of the hyaluronic acid micelles decreases the cellular uptake in vitro. Nevertheless, and more importantly, the cellular uptake was higher in the overexpressing CD44 tumor cells (SCC7, MDA-MB-231, and HCT11) than in the normal fibroblast cells (CV-1). The ex vivo images of the mice’s organs showed that the PEGylation of the hyaluronic acid micelles contributes to a significant decrease to their uptake by the liver, increasing their blood time circulation. Tests in animal models showed that, after systemic administration, the PEG-conjugated hyaluronic acid micelles were more effectively accumulated in the tumor when compared with the hyaluronic acid micelles. Driven by this result, the same group used the micelles as DDS for CPT [110]. The results evidence that the micelles were capable of quickly releasing the drug in the presence of Hyal-1, an enzyme that is found in the intracellular compartments of cancer cells. Regarding in vitro cytotoxicity tests performed on different tumor cell lines, they showed that, in some cases, the CPT-loaded micelles were more cytotoxic than free CPT. However, for non-cancerous cells, the cytotoxicity of the micelles was significantly lower than that of CPT. The authors attributed this result to the fact that the CPT-loaded micelles are specifically uptaken by the tumor cells via receptor (CD44)-mediated endocytosis, and the drug is quickly released at the intracellular level. The in vivo tests demonstrated that, after the systemic administration of the micelles in tumor-bearing mice, an inhibition of the tumor growth was observed.

Another attractive strategy for cancer therapy is the preparation of micelles based only on natural polymers. Na et al. [111] prepared micelles using amphiphilic copolymers based on glycol chitosan grafted with different amounts of 5β-cholanic acid. It was found that neither the size nor the shape was influenced by the amount of 5β-cholanic acid. However, the stability and deformability of the micelles decreased upon an increase in the amount of the acid. From the in vivo studies carried out on flank tumor-bearing mice, it was figured out that the micelles showed higher tumor accumulation when compared with glycol chitosan or polystyrene nanoparticles. The micelles with the highest targeting tumor efficiency were those obtained from the copolymer bearing 23 % of 5β-cholanic acid. The authors attributed this result to the good balance between the deformability and stability of the micelles in vivo.

In the same manner, very recently, Thomas et al. [112] prepared micelles from hyaluronic acid grafted with 5β-cholanic acid with the aim of developing DDS with targeting properties to the CD44 overexpressing cells. For micelles formulated with a PTX, hyaluronic acid 10 % *w*/*w*, the encapsulation efficiency was about 77 %, with a PTX loading of 7.7 %. The SCC7 cell line, which expresses the CD44 receptor, was used to evaluate the cytotoxicity of the PTX-loaded micelles, and the results highlight that the cell viability decreased significantly with an increase in the micelles’ concentration, when compared to the free drug. The in vivo tests revealed that the loaded micelles inhibited in a more effective way the tumor growth, with minimal side effects, when compared with free PTX.

#### Implants

**Fig. 15:**
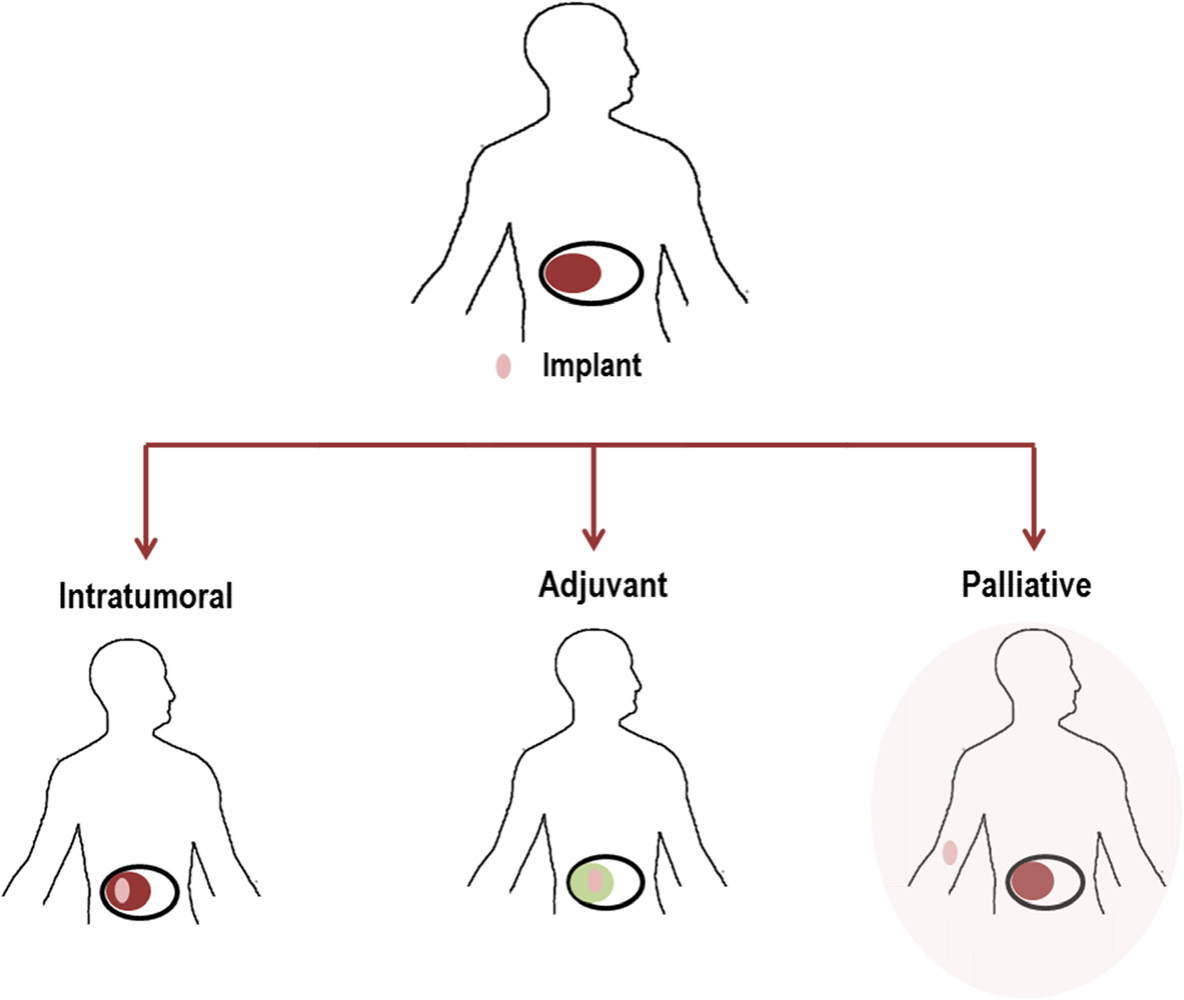
Different categories of the implants (adapted from [114])

Implants are very useful in cancer therapy since they promote the delivery of the drug in the “right” place and can provide the drug release over longer periods of time. This fact avoids the repeated external drug administration, increasing the patient’s compliance. The implants can be classified in surgical implants (pre-formed implants like rods, wafers, meshes), if they have a specific structure before the implantation, or in injectable implants [24, 113]. The polymer implants can be grouped into three main categories: intratumoral, adjuvant, and palliative (Fig. 15). Intratumoral implants are those that are placed directly into the tumor. If the implant is placed in the tumor after another treatment (e.g., surgery resection or ablation), it is called adjuvant. Palliative implants are those that are implanted subcutaneously or intramuscularly and are usually used to avoid the repeated injections and improve patient compliance in the late stage of palliative care [114].

There are already some polymer-based implants in the market or are under clinical trials, as can be seen in Table 2.

**Table 2:** Polymer-based implants used in the treatment and prevention of cancer (adapted from [24])

Polymer	Drug	Implant form	Trade name	Administration	Therapeutic purpose	Stage of development
Poly(anhydride)^a^	Carmustine	Wafer	Gliadel® Wafer	Intracavity resection	Glioblastoma brain cancer	Used clinically to treat glioblastoma brain cancer
Polyphosphoester	PTX	Microparticles	Paclimer®	Intraperitoneal	Advanced ovarian cancer	Phase I dose escalation trial for treatment of advanced ovarian cancer
PLGA-*b*-PEG-*b*-PLGA	PTX	Thermosensitive hydrogel	OncoGel™	Intratumoral	Esophageal cancer	Phase IIb efficacy trials for treatment of esophageal cancer

^a^Poly[(1,3-bis-carboxyphenoxypropane)-co-(sebacic anhydride)]

For localized therapy, besides the PLGA-*b*-PEG-*b*-PLGA, chitosan is another material extensively used in the development of injectable hydrogels for cancer therapy [115, 116]. Nevertheless, there is the need to perform more studies in order to release chitosan-based implants in the market.

Millirods are another type of implants potentially usable in the cancer field and have the main function of reducing the local recurrence rates of tumors when those are subjected to radiofrequency ablation. PLGA has been widely used in the preparation of millirods for this purpose [117–119]. In another approach, PLGA millirods were developed as DDS for the treatment of glioblastoma multiforme (GBM) via stereotactic injection [120]. The millirods were processed by two different techniques, viz., hot melt extrusion (HME) and injection moulding (IM), and loaded with disulfiram (DSF). The results showed that the processing technique influenced the physical state of DSF in the millirod; in the HME millirod, the drug was completely amorphous, whereas in the IM millirods, about 54 to 66 % of the DSF crystallinity was preserved. The in vitro cytotoxicity tests, using a GBM cell line, showed that the 10 wt.% DSF-loaded HME millirods presented cytotoxicity similar to free DSF. PLGA in the form of wafers was also tested as DDS with the same purpose [121].

In a very recent contribution, Gao et al. [122] developed an injectable implant based on PLGA, incorporating Fe powder and DOX that exhibited a liquid-solid transition when in contact with aqueous solutions. The in vitro release tests showed that the DOX release was accelerated in a pH = 5 and when the implant was subjected to an external alternating current magnetic field (AMF). In turn, the in vivo tests showed that, upon application of the AMF, the implant had the ability to convert magnetic energy into heat, increasing the local temperature in order to achieve tumor coagulative necrosis. The increase in temperature also contributed to accelerate the DOX release, improving the tumor cells’ apoptosis. The real-time ultrasound and computerized tomography imaging proved that no leakage of Fe particles occurred.

Other contributions report the use of bioabsorbable polymers like PCL or gelatin in the development of implants. Yohe et al. [123] prepared 3D superhydrophobic electrospun meshes based on PCL and the hydrophobic polymer dopant poly(glycerol monostearate-*co*-ε-caprolactone) (PGC-C18) and tested their potential as local DDS for colorectal cancer cells, using CPT-11 and SN-18 as model drugs. The release of the drugs can be modulated by changing the hydrophobicity of the mesh; a slower release is observed for more hydrophobic meshes. The in vitro cytotoxicity tests carried out on a human colorectal (HT-29) cell line showed that the SN-38-loaded meshes were cytotoxic for the cells, contrarily to what was observed with CPT-11-loaded meshes. Regarding the mechanical performance of the meshes, it was found that these structures are robust and flexible making them potentially usable in surgical procedures during colorectal surgery. Moreover, meshes allow the non-invasive assessment of their location and the status of drug delivery by ultrasound.

Recently, in a very interesting approach, Yang et al. [124] prepared an implantable active-targeting micelle-in-nanofiber device to be used as DDS for DOX, by the co-axial electrospinning method. The nanofibers’ core was formed by a mixture of poly(vinyl alcohol) and micelles (made from a folate-conjugated PCL-PEG copolymer), and the shell comprised crosslinked gelatin. The in vitro cytotoxicity tests showed that the device is non-cytotoxic either for 4T1 tumor cells or for NIH-3T3 fibroblasts. The anti-tumor efficacy was evaluated in 4T1 tumor cells, and the results showed that the nanofibers cause an apoptotic response for the 4T1 cell population of 69.9 %, this percentage being lower than that observed for the cell group treated with free DOX. The anti-tumor efficacy was tested with 4T1-tumor-bearing Balb/c mice, and the results showed that after 21 days, the mean volume of the tumors treated with the nanofibers is smaller than that in which DOX was delivered intravenously. However, the histological analysis confirmed that the DOX-loaded nanofibers induced apoptosis of the cancer cells.

## Conclusions

The importance of bioabsorbable polymers in the field of cancer therapy is undeniable, taking into account the high number of publications related to the use of these materials as DDS for anti-cancer drugs. Moreover, there are already in the market formulations for chemotherapy treatments making use of bioabsorbable polymers, and many others are currently under clinical trials. Regarding the fundamental research, many efforts have been devoted to the development of the ideal polymeric DDS, and although promising results have been obtained, important research efforts will have to be undertaken in order to develop optimal and efficient target DDS for cancer therapy.

## Abbreviations

AMF: alternating current magnetic field
ANG: angiopep-2
AuNPs: gold nanoparticles
BA: betulinic acid
BBB: blood brain barrier
BMECs: brain microvascular endothelial cells
BSA: bovine serum albumin
CMC: critical micelle concentration
CPT: camptothecin
DDS: drug delivery systems
DOX: doxorubicin
DPPE: 1,2-dipalmitoyl-sn-glycero-3-phosphoethanolamine
DSF: disulfiram
DTX: docetaxel
ɛ-CL: ɛ-caprolactone
EGFR: epidermal growth factor receptor
EPR: enhanced permeability and retention
FDA: Food and Drug Administration
FITC: fluorescein isothiocyanate
GA: glycolic acid
GBM: glioblastoma multiforme
GSH: glutathione
HME: hot melt extrusion
HSA: human serum albumin
IM: injection moulding
IP: isoelectric point
LA: lactic acid
miRNA: micro ribonucleic acid
MPEG-b-(PLA-co-PAE): poly(ethylene glycol) methyl ether-*b*-(poly(lactic acid-*co*-poly(β-amino ester)))
MPS: mononuclear phagocyte system
MRI: magnetic resonance imaging
OHB: oligo(3-hydroxybutyrate)
OHBHHx: oligo(3-hydroxybutyrate-co-3-hydroxyhexanoate)
PBEP-*b*-PBYP-*g*-PEG: poly(2-ethylbutoxy phospholane)-*b*-poly(2-butynylphospholane)-*g*-poly(ethylene glycol)
PCL: poly(ɛ-caprolactone)
PDLA: poly(D-lactic acid)
PDLLA: poly(D,L-lactic acid)
PEEP: poly(ethyl ethylene phosphate)
PEG: poly(ethylene glycol)
PEO-PHB-PEO: poly(ethylene oxide)-*b*-poly(3-hydroxybutyrate)-*b*-poly(ethylene oxide)
PGC-C18: poly(glycerol monostearate-*co*-ε-caprolactone)
P-gp: P-glycoprotein
PHA: poly(hydroxyalkanoate)
PHB: poly(3-hydroxybutyrate)
PHB4HB: poly(3-hydroxybutyrate-*co*-4-hydroxybutyrate)
PHBHHx: poly(3-hydroxybutyrate-*co*-3-hydroxyhexanoate)
PLA: poly(lactic acid)
PLGA: poly(lactic-co-glycolic acid)
PLLA: poly(L-lactic acid)
PTX: paclitaxel
PβAE: poly(β-amino ester)
RES: reticuloendothelial system
RFP: red fluorescence protein
RGD: arginylglycylaspartic acid
RHAMM: receptor for hyaluronan-mediated motility
ROP: ring opening polymerization
SCK: shell crosslinked knedel
siRNA: small interfering ribonucleic acid
SPIO: superparamagnetic iron oxide
SPION: superparamagnetic iron oxide nanoparticle
Tf: transferrin
VEGFR: vascular endothelial growth factor receptor
